# Progressive Changes in Commercial Orthopedic Drill Bits During Repeated Drilling of Synthetic Bone: Drill-Point Geometry, Thrust Force, Feed Work, and Hole Diameter

**DOI:** 10.3390/ma19194042

**Published:** 2026-09-22

**Authors:** Tomasz Bartkowiak, Mikołaj Nęcka, Marcin Pelic, Paweł Zawadzki, Dominika Podbereska, Stanisław Pabiszczak, Michał Jakubowicz, Jakub Czyżycki, Paweł Twardowski, Jakub Stefaniak, Maciej Szymankiewicz, Jakub Głowacki

**Affiliations:** 1 Institute of Mechanical Technology, Poznan University of Technology, 61-131 Poznan, Polandmarcin.pelic@put.poznan.pl (M.P.); pawel.zawadzki@put.poznan.pl (P.Z.); stanislaw.pabiszczak@put.poznan.pl (S.P.); michal.jakubowicz@put.poznan.pl (M.J.); jakub.czyzycki@put.poznan.pl (J.C.); pawel.twardowski@put.poznan.pl (P.T.); 2 Rehasport Clinic, 60-201 Poznan, Poland; jakub.stefaniak@rehasport.pl; 3 Poznan University of Medical Sciences Poznan, Adult Spine Orthopaedics Department, 61-545 Poznan, Poland; 4 Poznan University of Medical Sciences, Department of Spine Disorders and Pediatric Orthopedics, 61-545 Poznan, Poland; dr.jakubglowacki@gmail.com

**Keywords:** orthopaedic drilling, repeated drill use, drill condition, thrust force, axial feed work, projected point angle, hole diameter, synthetic bone

## Abstract

Repeated use can alter the geometry and functional performance of orthopedic drill bits, yet geometry, process load, and hole dimensions are rarely tracked together. Four integrated commercial drill types—Stryker (3.2 and 3.5 mm), Synthes (4.5 mm), and Zimmer (4.3 mm)—produced successive through-holes in a 6 mm short-glass-fiber-filled epoxy cortical-bone analog at 900 rpm and approximately 40 mm/min. Projected point angle was measured intermittently, maximum thrust force and axial feed work were calculated per hole, and entrance diameter was measured optically. The primary estimand was longitudinal change within each drill type; the design was not factorial, and differences among types cannot be attributed separately to manufacturer, diameter, geometry, intended function, or alloy. Drill-type-specific models included fixed specimen intercepts. Force, work, and diameter were described using constrained monotonic functions selected by AICc, while trend inference used permutation-based Spearman tests. Point angle decreased significantly only for the Stryker drills. Force and work increased in every type, whereas entrance diameter decreased significantly for Stryker 3.5 mm, Synthes, and Zimmer. Pairwise force/work–diameter associations did not remain significant after adjustment for hole number and multiplicity. Repeated-use exposure therefore produced drill-type-specific mechanical and dimensional drift, but the study did not directly quantify cutting-edge wear or establish a causal material mechanism.

## 1. Introduction

Bone drilling is a routine but mechanically demanding step in fracture fixation, plate osteosynthesis, and intramedullary procedures. A clinically acceptable pilot hole must follow the intended trajectory and dimensions while limiting mechanical and thermal injury to the surrounding tissue. This balance is difficult to maintain because bone is heterogeneous and anisotropic: mineralization, porosity, moisture, anatomical orientation, and cutting direction all influence its response. Consequently, force and temperature can vary even when the nominal drilling parameters remain unchanged [1,2,3,4].

Drill condition adds another source of variability. Repeated use can round the cutting lips and chisel edge, abrade the margins, and produce localized microdamage. As sharpness declines, a greater fraction of tool action may involve plowing, compression, and friction rather than efficient cutting, increasing mechanical and thermal demand [2,3,4,5]. However, in this study, edge radius, wear land, worn area, and material loss were not measured directly. The number of holes is therefore treated as an exposure index, and the term wear is used only as a literature-based interpretation of repeated-use-related changes rather than as a directly measured quantity. Commercial drills may also differ in initial geometry, alloy, heat treatment, surface finish, coating, and manufacturing quality [6,7,8].

Systems used to assess orthopedic drill wear can be divided broadly into direct and indirect approaches. Direct systems interrupt drilling to inspect or measure the tool itself. Optical and digital microscopy have been used to quantify wear area, point-angle change, chisel-edge widening, deformation, and the loss of manufacturing marks; scanning electron microscopy provides higher-resolution evidence of edge rounding, microchipping, grooves, and adhered debris [9,10,11]. Other studies have supplemented imaging with profilometry, surface-roughness measurements, or gravimetric mass loss, thereby describing changes that may not be visible in a single two-dimensional view [12]. These methods provide physically interpretable evidence of damage, but they are usually intermittent and sensitive to imaging orientation, magnification, illumination, and the selected region of the cutting edge.

Indirect systems infer drill condition from the response of the cutting process. Piezoelectric or multiaxis dynamometers are commonly used to record thrust force and torque, while thermocouples or infrared thermography quantify the thermal consequence of repeated drilling [7,8,10,13,14]. More recent monitoring platforms combine triaxial force with vibration, servomotor current, or acoustic-emission signals and use extracted features or machine learning classifiers to distinguish tool-condition states [15,16,17]. Such systems can operate during drilling and are attractive for automated or robotic procedures, although their outputs remain indirect: force, vibration, and temperature are also affected by feed, speed, drilling depth, chip evacuation, substrate properties, and tool geometry [3,4,18,19].

Direct inspection and process monitoring answer complementary questions. Imaging can show how the drill changes, whereas sensor signals show whether cutting performance changes. Experimental studies have linked spindle speed, feed rate, diameter, point and helix geometry, irrigation, and drill condition to force, torque, temperature, and hole quality [3,4,18,19,20,21,22,23,24,25,26,27,28,29,30,31,32]. Synthetic materials improve control and repeatability, ex vivo bone improves biological realism, and finite-element models help separate thermal and mechanical contributions, but none of these approaches alone reproduce the complete clinical process [27,28,33,34,35,36]. This diversity also means that force or temperature values should not be transferred between studies without considering substrate and drilling conditions.

A standardized synthetic substrate supports this type of longitudinal comparison by reducing specimen-to-specimen variability. Its response, however, cannot be assumed to reproduce the human cortex. Direct comparisons have demonstrated substrate-dependent differences in force and torque between artificial and human femurs and among artificial, bovine, and porcine femoral materials [35,36]. Results obtained in synthetic bone should therefore be interpreted as controlled comparative evidence rather than as direct estimates of clinical load.

Dimensional accuracy is potentially clinically relevant because the opening becomes a pilot hole for screw insertion. Previous studies show that pilot-hole diameter can influence insertion torque and fixation mechanics [37,38,39]. A smaller opening may increase interference and thread-forming demand, with possible consequences for compaction, microdamage, pullout resistance, or instrument failure. These consequences were not measured in the present study and are discussed only as hypotheses requiring direct screw-insertion and fixation testing.

The primary objective was to quantify longitudinal changes in projected point angle, maximum thrust force, axial feed work, and entrance-hole diameter within each integrated commercial drill type during repeated drilling under one controlled condition. A secondary objective was to examine whether associations among these responses persisted after accounting for their common progression with hole number. Because the manufacturer, nominal diameter, geometry, intended function, material, and exposure range differed simultaneously, this study was not designed to isolate any one factor or to rank manufacturers. It was hypothesized that repeated-use exposure would change mechanical demand and hole dimensions, while the magnitude and trajectory would depend on the complete drill configuration.

## 2. Materials and Methods

### 2.1. Experimental Design, Drills, and Test Material

This study used Sheet, Absolute™ 4th Gen synthetic cortical-bone analog (Sawbones, Pacific Research Laboratories, Vashon Island, WA, USA; SKU 3401-04), supplied as 130 mm × 180 mm × 6 mm sheets. The manufacturer identifies the material as a short-glass-fiber-filled epoxy intended to simulate cortical bone for structural biomechanical testing [40]. Four physical drills of each type were tested. Stryker drills produced 20 holes per tool, whereas Synthes and Zimmer drills produced 60 holes per tool.

The four commercial drill configurations were Stryker 3.2 mm and Stryker 3.5 mm drills manufactured by Stryker GmbH (Selzach, Switzerland), a DePuy Synthes 4.5 mm drill manufactured by Synthes GmbH (Oberdorf, Switzerland), and a Zimmer Biomet 4.3 mm drill manufactured by Zimmer Switzerland Manufacturing GmbH (Winterthur, Switzerland). All drill bits were purchased in 2025. Catalogue identifiers were verified against the available product labels. Table 1 summarizes their nominal dimensions, product designations, couplings, and flute information; product documentation and an authoritative device record provide additional context [41,42,43,44].

Manufacturer-specified point angles, helix angles, web thicknesses, cutting-edge radii, and coating specifications were not established from the available documentation and were not assigned assumed values. Projected drill-point angles were measured experimentally using the optical silhouette method (Section 2.2), with the results reported in Section 3.1. These projected measurements should not be equated with a manufacturer-specified three-dimensional point geometry. Elemental composition was assessed separately by EDXRF (Section 2.6). No apparent coating was visible.

The nominal diameters reflected the selected commercial configurations used in orthopedic fixation instrument sets. This study was designed to characterize their longitudinal response to repeated drilling under one controlled condition; it was not a factorial experiment separating diameter, manufacturer, geometry, or material. Product function also differed: the Stryker 3.5 mm instrument is an overdrill intended to prepare a near-cortex gliding hole for lag-screw fixation [42]. Accordingly, drill type denotes the complete commercial configuration, including its intended function, and the contribution of any individual design or material feature cannot be isolated.

The unequal exposure windows arose during protocol development. A preliminary qualitative series used two drills and showed significant changes of geometry in the Stryker drills. On that basis, 20 holes was adopted as a pragmatic stopping limit for Stryker, whereas Synthes and Zimmer were continued to 60 holes. Direct between-type comparisons and comparisons of model form across unequal exposure ranges were therefore excluded.

A new location was used for every hole. Holes were drilled in a linear arrangement, with a minimum edge distance of 2 mm, which avoided interactions between subsequent holes. No hole intersected a previous hole or the sheet edge. Point-angle measurements were taken before drilling (hole number 0) and at predefined stages; force was recorded for every event, and hole diameter was measured once after drilling for every hole.

### 2.2. Vision-Based Measurement of Projected Drill-Point Angle

For point-angle imaging, each drill was positioned between a Basler ace digital camera (Basler AG, Ahrensburg, Germany) fitted with a microscope objective and a red backlight, producing a high-contrast silhouette. Exposure and gain were fixed to suppress reflections. Image analysis was implemented in Python using the Open Source Computer Vision Library (OpenCV; https://opencv.org/; accessed on 16 September 2026). A representative image of a drill tip recorded by the camera is shown in Figure 1.

Images were smoothed with a 41 × 41 pixel Gaussian kernel (σ = 25). Only the red channel was retained and binarized at 90/255, followed by morphological closing with a 17 × 17 pixel structuring element. The silhouette was sampled over 1200 image columns; upper and lower boundaries were extracted, and missing or isolated outlying contour points were removed. Images with insufficient valid contour information were rejected.

The apex-adjacent contour was not fitted as a single line because rounding or chipping can locally distort it. Multiple candidate segments were created independently for the upper and lower edges by holding the right boundary fixed and progressively shifting the left boundary. Linear regression was fitted to each candidate. Segments shorter than 40% of the available contour were rejected, and the retained segments were scored using goodness of fit, slope, length, and location. The highest-scoring segment represented each edge. For line slopes *m*_1_ and *m*_2_, the acute angle between the lines was calculated from *arctan*(|(*m*_2_ − *m*_1_)/(1 + *m*_1_*m*_2_)|), and line orientation was used to obtain the internal projected point angle. The result is a two-dimensional apparent angle in the defined imaging plane.

The image-acquisition protocol did not include replicate removal and repositioning of the same drill. A formal measurement-system repeatability or uncertainty estimate is therefore unavailable. Sub-degree changes, particularly for Synthes and Zimmer, are interpreted as no statistically detectable change under this protocol rather than as evidence that no geometric change occurred.

### 2.3. Drilling Procedure

The drilling station is shown in Figure 2. Drilling was performed on an AVIA FNC-25 milling machine (AVIA, Warsaw, Poland) at 900 rpm and an approximately 40 mm/min programmed feed, corresponding to 0.044 mm/rev. These settings were selected during protocol development to provide stable through-drilling and a measurable force signal and lie within the low-to-moderate parameter ranges represented in the experimental orthopedic-drilling literature [1,2,3,4,22,23]. They were held constant to isolate repeated-use exposure; the experiment was not intended to optimize speed or feed or to estimate their interaction. The 6 mm sheet was drilled through under constant axial feed.

All four configurations, including the Stryker 3.5 mm overdrill, were evaluated using this same through-drilling protocol. The experiment therefore characterizes their responses under the stated bench conditions and does not reproduce every instrument’s intended clinical use.

### 2.4. Thrust-Force Measurement and Signal Processing

The thrust force generated during drilling was measured using a three-component piezoelectric dynamometer installed between the specimen fixture and the machine table. The charge signal generated by the dynamometer was conditioned and amplified using a Kistler 5015 charge amplifier (Kistler, Winterthur, Switzerland). The amplified signal was digitized using an analog-to-digital converter and transmitted to a computer. Force–time signals were recorded using proprietary Analizator software (version 2020.01; created by Prof. Maciej Tabaszewski). The force component parallel to the drill axis, (F_z_), was treated as the thrust force.

Force signals were sampled at 5 kHz. For each trace, the drilling interval was isolated and baseline-corrected. Rotational components were attenuated using notch filters at the 15 Hz spindle fundamental (900 rpm/60) and harmonics, with Q = 30. An 8 Hz low-pass filter was then applied. The 8 Hz cutoff was selected to retain the slowly varying force envelope associated with constant axial feed while suppressing rotational content above the response band used to define the drilling-load trend. Because a maximum is sensitive to short transients, all conclusions were checked against unfiltered signals. Maximum thrust force, Fmax, was the maximum of the filtered interval. Axial feed work was calculated as *W = v_f_∫F*(*t*)*dt = ∫F dz*, using *v_f_ =* 40 mm/min; it represents work done by the axial feed force, not spindle-cutting energy. Filtering changed median *F_max_* by −5.68% and median work by 0.001%, and raw- and filtered-signal conclusions were concordant.

### 2.5. Optical Measurement of Hole Diameter

Hole diameters were measured using a Hexagon Optiv Reference 763 Dual Z multisensor coordinate measuring machine (Hexagon Manufacturing Intelligence, Stockholm, Sweden) with a fixed-bridge granite structure and two independent vertical sensor axes. Although the system supports vision, tactile scanning, and white-light interferometric sensing, only vision was used. The optical system comprised two 752 × 582 pixel monochrome cameras with up to 5× optical magnification, multiple illumination modes, active vibration damping, temperature compensation, and PC-DMIS CAD++ software (Hexagon Manufacturing Intelligence; https://hexagon.com/products/pc-dmis; accessed on 16 September 2026).

After removal of loose debris without modifying the hole edge, plates were thermally stabilized and measured at 20 ± 0.3 °C on the transparent measurement table. The upper plate surface defined the primary datum. Transmitted bottom/profile illumination produced a high-contrast aperture boundary, and autofocus established the focal position at the upper surface. The Auto Feature Circle function defined an annular region of interest and detected the material–air edge using consistent magnification, illumination, and edge settings. A least-squares-associated circle was fitted to the detected circumference. The measurand was the entrance diameter at the upper plate surface, and each hole was measured once.

### 2.6. Chemical Composition of the Drills

The elemental composition of the drill bits was determined by energy-dispersive X-ray fluorescence spectrometry (EDXRF) using a FISCHERSCOPE^®^ X-RAY XDV-SDD spectrometer (Helmut Fischer GmbH, Sindelfingen, Germany). The measurements were performed at a tube voltage of 50 kV and an anode current of 1000 µA, using a 0.60 mm collimator and an Ni 10 primary filter. The acquisition time for each spectrum was 60 s.

Five measurements were performed for each drill specimen (*n* = 5) at different areas of its surface. The measurements were evaluated using the standard-free Fe–Cr–Mo–Ni–Mn analytical application. The contents of the determined elements were expressed as mass percentages (wt.%).

The available analytical configuration did not permit the quantification of carbon. Therefore, carbon was not included in the reported chemical compositions, and its absence from the results should not be interpreted as the absence of carbon from the investigated alloys. Consequently, the measured elemental profiles allowed only tentative assignment of the drills to particular stainless steel families. Grades differing primarily in carbon content, particularly AISI 440A, 440B, and 440C, could not be distinguished conclusively.

The stainless steel designation on the Synthes and Zimmer product labels is a broad material description, not a certificate of alloy grade, hardness, or heat treatment. These label declarations were kept separate from the exploratory EDXRF-based compositional similarities.

### 2.7. Statistical Analysis

Analyses were performed separately for each drill type; force and axial feed work were not compared between types because nominal diameter, geometry, and intended function differed. For physical drill *i* and hole/stage *j*, the descriptive model was *y_ij_* = *α_i_* + *g*(*h_j_*;*θ*) + *ε_ij_*, where α_i_ is a fixed specimen-specific intercept and *g* is the type-level exposure function. Candidate functions were linear, logarithmic, and asymptotic exponential; coefficients were constrained to make force/work non-decreasing and diameter non-increasing. The lowest-AICc model was used for descriptive estimation, and confidence bands were obtained by delta-method propagation of the parameter covariance matrix. Fixed rather than random intercepts were used because only four known drills were available per type; estimating a between-drill variance component and serial-correlation structure from four clusters would be unstable and would rely on unverifiable asymptotic assumptions. The nonlinear model curves were descriptive and were not used as the primary source of trend *p*-values.

Statistical evidence for monotonic change in force, work, and diameter was based on Spearman’s *ρ* calculated from drill-type means at complete hole-number stages, with exact or 200,000-repetition Monte Carlo permutation tests and Holm correction across drill types within each response. This stage-mean analysis was selected to avoid treating the many holes from the same physical drill as independent replicates. Each plotted point is the arithmetic mean of the four physical drills at that hole or common stage; the shaded bands are 95% confidence intervals for the fitted type-level mean response. Separate pointwise error bars were not added because the between-drill component was already represented by specimen-specific intercepts and model covariance, whereas an error bar based on four values could be mistaken for measurement uncertainty. Individual observations were not plotted to preserve legibility and because the prespecified estimand was the type-level longitudinal trajectory.

Pairwise relationships (force–diameter, work–diameter, force–angle, work–angle, and angle–diameter) were first summarized by Spearman’s *ρ* for type means at common stages. Partial within-type associations were then estimated in models containing hole number and fixed physical-drill indicators, with HC3 inference, to test whether pairwise association persisted beyond shared progression with repeated use. Correlation *p*-values were Holm-adjusted. Complete-case matching was used, so analyses involving point angle contained fewer stages than force–diameter analyses. With only four drill sequences per type, an additional random-effect plus serial-correlation model was not considered reliably identifiable; residual within-series dependence remains a limitation.

## 3. Results

### 3.1. Projected Drill-Point Angle

The image sequences in Figure 3 provide a qualitative overview of drill-point evolution. Synthes 4.5 mm and Zimmer 4.3 mm retained a broadly similar macroscopic outline through hole 20, whereas Stryker 3.2 mm changed modestly and Stryker 3.5 mm showed pronounced flattening. Figure 4 provides a worked calculation: candidate contour segments were fitted independently on the two flanks, the selected slopes were inserted into the stated line-angle equation, and line orientation selected the internal projected angle. For the representative new Synthes drill, this produced 67.07°, with R^2^ values of 0.9989 and 0.9992 for the two fitted flanks. These images do not quantify edge radius, worn area, or material loss.

Point-angle evolution differed among drill types (Figure 5). For Stryker 3.2 mm, the linear model estimated a decrease from 84.30° to 80.67° over 20 holes (−3.63°, −4.31%; HC3 *p* < 0.001). Stryker 3.5 mm showed a larger nonlinear decrease, from 75.11° to 51.96° (−23.15°, −30.82%; *p* < 0.001). The modeled changes for Synthes 4.5 mm (−0.16°, *p* = 0.416) and Zimmer 4.3 mm (−0.54°, *p* = 0.437) were not statistically detectable. Their apparently discrete trajectories reflect intermittent stage measurements combined with subdegree mean changes and between-stage scatter. In the absence of a formal repositioning-repeatability study, these nonsignificant results are not interpreted as proof of geometric stability.

### 3.2. Maximum Thrust Force and Axial Feed Work

Filtered maximum thrust force increased monotonically with hole number in every drill type (Holm-adjusted permutation *p* ≤ 0.0016; Figure 6). Asymptotic–exponential models were selected for Stryker 3.2 and 3.5 mm, indicating rapid initial growth followed by a plateau within the observed 20-hole window. Logarithmic models were selected for Synthes and Zimmer over their 60-hole windows, indicating continued but decelerating growth. These model forms describe separate exposure ranges and were not compared as evidence of superior performance. Modeled first-to-last increases were +58.92 N for Stryker 3.2 mm, +103.60 N for Stryker 3.5 mm, +71.63 N for Synthes 4.5 mm, and +68.39 N for Zimmer 4.3 mm; stage-mean Spearman coefficients ranged from ρ = 0.686 to 0.957.

Axial feed work also increased monotonically in every type (all Holm-adjusted *p* < 0.001). Asymptotic-exponential models were selected for Stryker 3.2 mm, Stryker 3.5 mm, and Zimmer 4.3 mm, whereas Synthes 4.5 mm was described by a logarithmic response. Modeled increases were +0.177 J, +0.285 J, +0.233 J, and +0.222 J, respectively. The co-increase in peak force and accumulated axial work is consistent with declining cutting efficiency during repeated use. Edge rounding, plowing, compression, friction, and impaired chip evacuation are plausible literature-consistent explanations, but they were not directly verified because edge radius and worn surface were not measured. These results are interpreted within type only.

### 3.3. Hole Diameter

Hole entrance diameter decreased significantly for Stryker 3.5 mm, Synthes 4.5 mm, and Zimmer 4.3 mm (Holm-adjusted permutation *p* ≤ 0.00018; Figure 7). Stryker 3.5 mm showed a modeled linear reduction of 0.185 mm; logarithmic reductions were 0.065 mm for Synthes and 0.045 mm for Zimmer. For Stryker 3.2 mm, an asymptotic model estimated a 0.029 mm reduction, but the monotonic stage-mean association was not significant (ρ = −0.135, adjusted *p* = 0.568). The isolated Synthes value of 4.218 mm at hole 42 was excluded during post-acquisition review. No prespecified exclusion rule or retained diagnostic record was available to verify the failure of the optical edge/circle fit. The observation was therefore not used in the trend model, and the exclusion is treated as a limitation rather than as an objectively confirmed measurement failure. The value and timing of the exclusion are reported here transparently.

### 3.4. Associations Among Force, Work, Point Angle, and Hole Diameter

At the drill-type mean level, maximum thrust force was negatively correlated with hole diameter for Stryker 3.5 mm (ρ = −0.676, Holm-adjusted *p* = 0.005), Synthes (ρ = −0.758, *p* < 0.001), and Zimmer (ρ = −0.550, *p* < 0.001), but not for Stryker 3.2 mm (Figure 8). Axial feed work showed the same unadjusted pattern, with significant negative correlations for Stryker 3.5 mm (ρ = −0.955), Synthes (ρ = −0.739), and Zimmer (ρ = −0.429), all adjusted *p* ≤ 0.0015 (Figure 9).

These associations largely reflected common progression with hole number. After adjustment for hole number and physical drill, none of the force–diameter or work–diameter partial associations remained significant after Holm correction (all adjusted *p* ≥ 0.086). Therefore, the data support parallel evolution during repeated use, but not an independent one-variable relationship in which force or work alone determines entrance diameter.

None of the force–angle, work–angle, or angle–diameter Spearman correlations were significant after Holm correction, and none of the run-adjusted partial associations was significant (Figure 10, Figure 11 and Figure 12). Large unadjusted coefficients in small Stryker subsets (e.g., ρ = −0.90 for force–angle in Stryker 3.2 mm and ρ = −1.00 for work–angle in Stryker 3.5 mm) were imprecise and did not survive exact multiplicity-adjusted testing. Accordingly, point angle alone was not supported as a surrogate for mechanical demand or dimensional output.

### 3.5. Chemical Composition of the Drills

EDXRF identified differences in the restricted Fe-Cr-Mo-Ni-Mn elemental profiles of the investigated drills (Table 2). The measurements characterize compositional differences among the tested commercial tools. They do not constitute a paired before-and-after analysis of chemical change during drilling and do not establish wear resistance. The stainless steel declarations for Synthes and Zimmer (Section 2.1) do not resolve their exact alloy grades. Stryker 3.2 mm, Synthes 4.5 mm, and Zimmer 4.3 mm showed high chromium and low nickel, whereas Stryker 3.5 mm showed lower chromium and higher nickel.

The Stryker 3.2 mm and Synthes 4.5 mm profiles were compositionally similar to martensitic stainless steels in the AISI 440 family. Because carbon—the principal discriminator among 440A, 440B, and 440C—was not measured, no specific 440 subtype is assigned.

The Zimmer 4.3 mm profile, including approximately 17.65% Cr and 1.00% Mo, was similar to X90CrMoV18/1.4112-type martensitic steel. This is an exploratory similarity only because carbon and vanadium were not quantified.

The Stryker 3.5 mm drill had a compositionally distinct restricted profile (11.19% Cr and 8.83% Ni). Within the measured elements, it was more similar to Custom 455/UNS S45500-type precipitation-hardening stainless steel than to AISI 304; however, copper, titanium, niobium, and carbon were not quantified. The alloy grade therefore cannot be identified definitively, and the profile is not used to explain the mechanical or dimensional trends causally.

The relatively small standard deviations observed for most elements indicated good repeatability of the measurements within each drill. However, because the XRF results were normalized to the analyzed Fe–Cr–Mo–Ni–Mn system, they should not be interpreted as complete bulk compositions. In particular, the absence of carbon from Table 2 does not indicate that the investigated steels were carbon-free.

## 4. Discussion

The principal finding was that repeated-use exposure changed functional performance and dimensional output in every drill type, whereas the projected point angle changed substantially only in the two Stryker groups. This divergence supports a multimodal assessment of drill condition. It does not demonstrate a universal wear mechanism: initial point, lip, chisel edge, web, flute, and margin geometry, together with alloy, heat treatment, surface condition, and runout, were confounded within each commercial type. The constrained models describe within-type trajectories and must not be interpreted as causal manufacturer or material effects.

The confirmed product identities also identify a difference in intended function. Stryker 703694 is a VariAx 2 overdrill for near-cortex gliding-hole preparation [42], whereas all tools in this experiment underwent the same through-drilling procedure. Its observed response must therefore be interpreted for this bench protocol. The present comparison does not isolate the influence of overdrill design or establish relative durability during the instruments’ respective clinical uses.

The increase in maximum thrust force is consistent with studies of sharp versus used drills and repeated drilling [5,7,8]. Edge rounding, increased plowing and compression, friction, impaired chip evacuation, and changing contact conditions offer plausible explanations [2,3,4,5,18,19], but none were directly verified here because edge radius, wear land, debris adhesion, torque, and vibration were not measured. The asymptotic and logarithmic functions should therefore be understood as empirical descriptions over different observation windows, not as proof of a specific damage mechanism or of tool recovery/stabilization beyond the tested range. The near invariance of axial feed work after filtering, compared with the modest reduction in Fmax, indicates that work was less sensitive to narrow high-frequency peaks.

EDXRF adds a limited compositional descriptor to the integrated drill-type comparison. It distinguishes a high-chromium/low-nickel group from the lower-chromium/higher-nickel Stryker 3.5 mm profile, but it cannot identify certified alloy grades or connect composition to repeated-use behavior. Carbon, vanadium, copper, titanium, and niobium were not quantified, and hardness, heat treatment, microstructure, surface condition, and residual stress were not measured. Accordingly, the elemental profiles are reported as exploratory similarities only.

These assignments must remain provisional. Carbon—the principal element used to distinguish AISI 440A, 440B, and 440C—could not be measured by the applied EDXRF configuration; vanadium was also unavailable for confirming X90CrMoV18, and copper, titanium, and niobium were not quantified to confirm Custom 455. More importantly, composition alone does not establish hardness, microstructure, carbide population, residual stress, aging condition, or surface treatment. Custom 455 is precipitation hardenable, and its hardness can change markedly with aging through the formation and evolution of nanoscale precipitates [45]. Previous studies of stainless steels used for medical and dental instruments likewise show that chemical composition alone does not characterize the resulting surface condition, hardness, or drill performance [46,47]. Consequently, the pronounced point-angle and diameter changes of the Stryker 3.5 mm drill cannot be attributed simply to a supposedly softer alloy. They may reflect the actual heat-treatment condition, initial edge geometry, local plastic deformation, surface finish, or an interaction among these factors. Direct hardness testing, metallography, and higher-resolution chemical analysis are required before a causal materials explanation can be established.

The decrease in entrance diameter demonstrates dimensional drift under fixed machine settings. Potential contributors include lip or margin recession, edge rounding, compression, elastic recovery, runout, vibration, and asymmetric tool change; the present measurements cannot distinguish among them. The disappearance of force/work–diameter associations after adjustment for hole number indicates parallel progression rather than a direct bivariate law.

Previous pilot-hole studies show that diameter can influence insertion torque and pullout behavior [37,38,39]. The undersizing observed here could therefore have implications for thread-forming demand, local compaction, microdamage, or fixation, but none of these outcomes were measured. The present study demonstrates dimensional drift only; any consequence for the screw–bone interface remains a hypothesis for dedicated insertion-torque, pullout, and damage experiments.

The absence of significant angle-based correlations does not mean that cutting geometry is unimportant. Projected point angle is a global two-dimensional measure and cannot capture edge radius, chisel-edge recession, lip-height asymmetry, margin wear, microchipping, or runout. Experimental and computational studies show that point angle, helix angle, web thickness, rake, margins, and cutting-edge arrangement can alter thrust force, processing energy, temperature, and surface integrity [19,24,31,32]. The measurement literature similarly indicates that no single sensing modality provides a complete description of wear: microscopy and three-dimensional optical metrology identify physical damage, while force, vibration, motor-current, and acoustic-emission systems reveal its functional consequences [10,11,15,16,17]. Combining these approaches with composition, hardness, and hole-metrology measurements offers a more defensible basis for drill-condition assessment.

This study has several limitations. It used one short-glass-fiber-filled epoxy substrate, one speed-feed combination, four physical drills per type, and unequal exposure windows. The drill types differed simultaneously in manufacturer, diameter, geometry, intended function, material, and surface condition, and several manufacturer design specifications were unavailable; consequently, only within-type longitudinal conclusions are supported. The substrate’s density, hardness, strength, and modulus were not available for the specific product and were not measured. Temperature, torque, vibration, three-dimensional hole geometry, edge radius, wear land, hardness, microstructure, insertion torque, microdamage, and pullout strength were not assessed. Point-angle measurements were intermittent, and no formal repositioning-repeatability study was performed. The fixed-intercept models and robust/permutation inference reduced reliance on fragile distributional assumptions, but four drill clusters were insufficient for a stable random-effect plus serial-correlation model. Future validation should use more tools, multiple drilling conditions, direct edge metrology, and biological bone; these extensions were beyond the scope and resources of the present study. One diameter observation was excluded after data review without a prespecified rule or retained diagnostic record, and sensitivity to that decision was not quantified.

## 5. Conclusions

Under one fixed drilling condition in a standardized synthetic cortical analog, repeated use increased maximum thrust force and axial feed work in all four integrated commercial drill types. Statistically detectable point-angle decreases occurred only in the Stryker groups, while entrance diameter decreased for Stryker 3.5 mm, Synthes 4.5 mm, and Zimmer 4.3 mm. Because the drill types differed simultaneously in manufacturer, diameter, geometry, intended function, material, and exposure range, these findings support within-type conclusions, not causal rankings of manufacturers or alloys.

The engineering implication is that neither a fixed hole count nor projected point angle alone is a sufficient condition-monitoring criterion. A practical replacement strategy should combine a process indicator such as force or axial feed work with dimensional verification of the produced hole and, after validation, temperature or direct-edge metrology. The observed undersizing may affect screw insertion, but this was not tested. EDXRF established only restricted elemental-profile differences and did not identify alloy grade or wear resistance. Clinical thresholds therefore require validation in biological bone with direct thermal, geometric, and fixation outcomes.

## Figures and Tables

**Figure 1:**
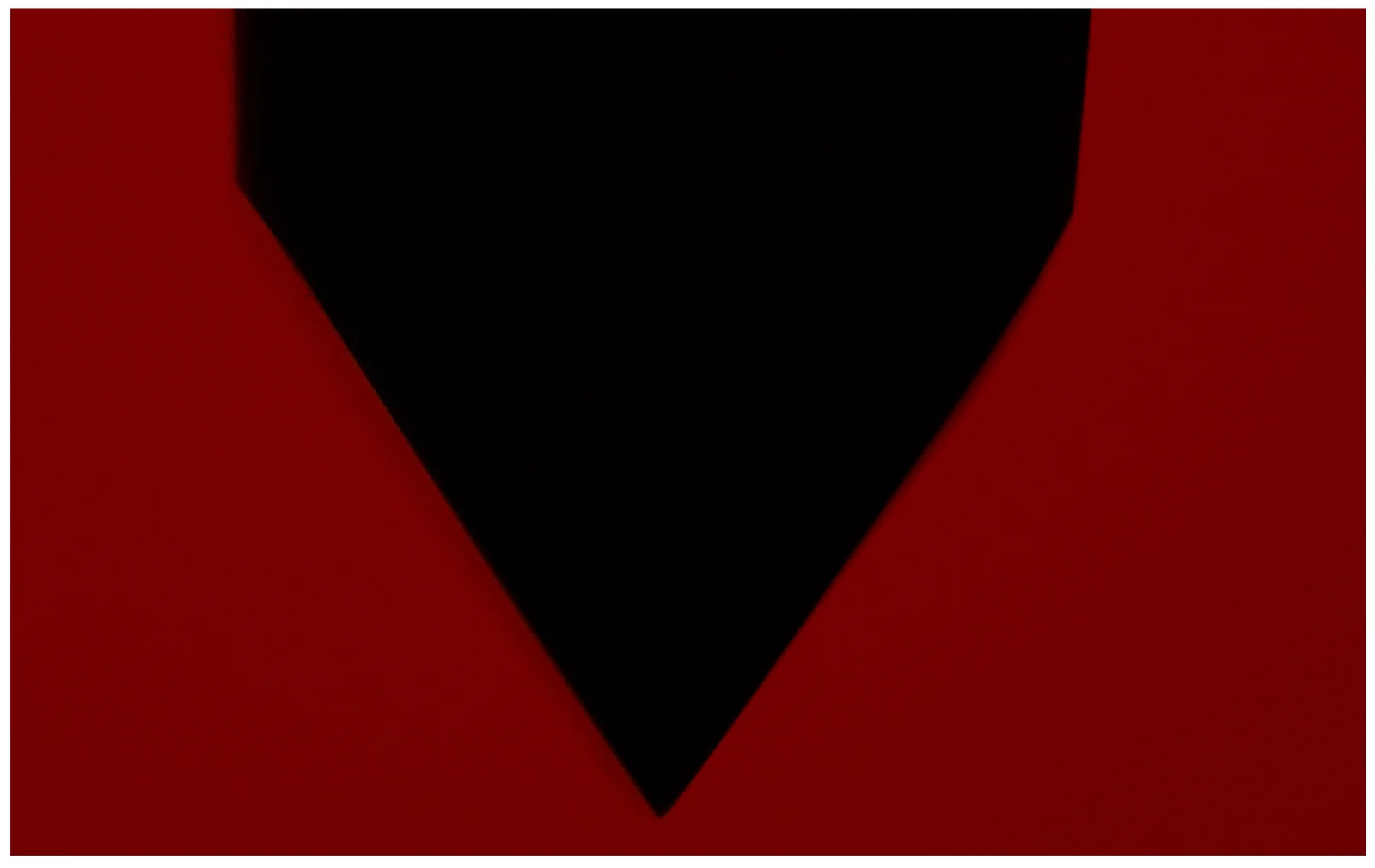
Representative image of a drill tip recorded with the vision system.

**Figure 2:**
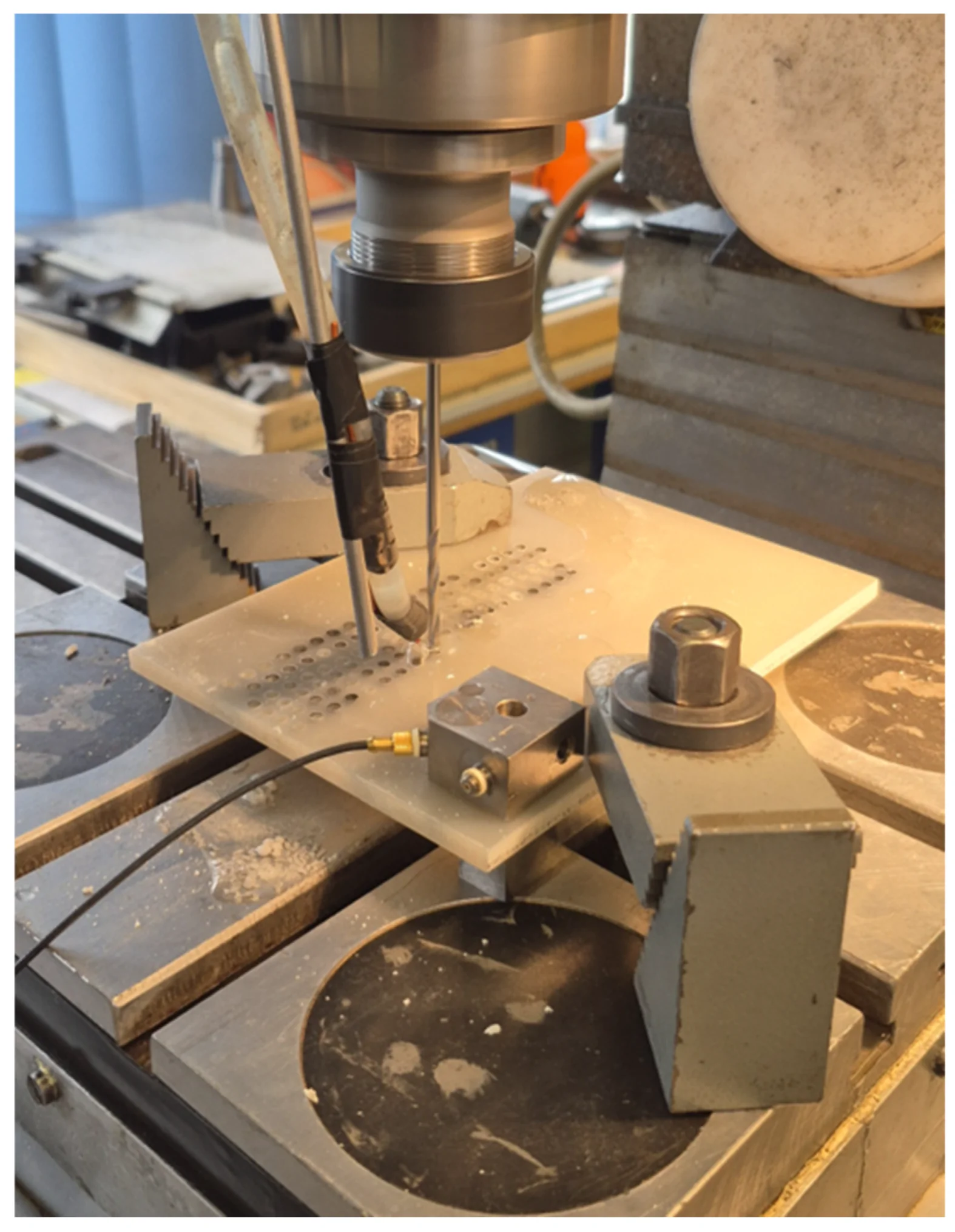
Photograph of the experimental drilling setup showing the spindle-mounted drill bit, the 6 mm synthetic cortical-bone surrogate, the specimen holder, and the force sensor.

**Figure 3:**
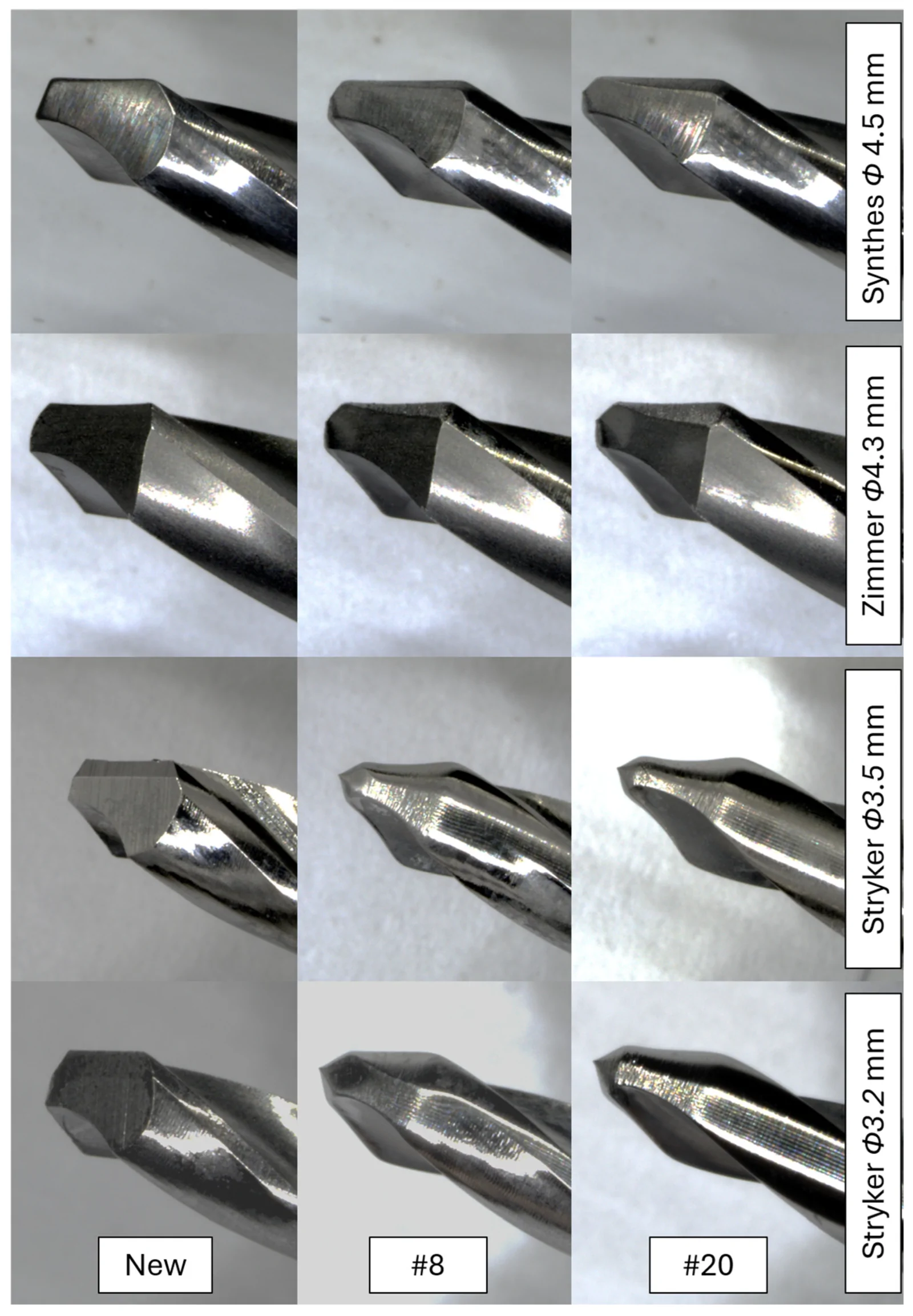
Representative drill-point images in the new condition and after holes 8 and 20.

**Figure 4:**
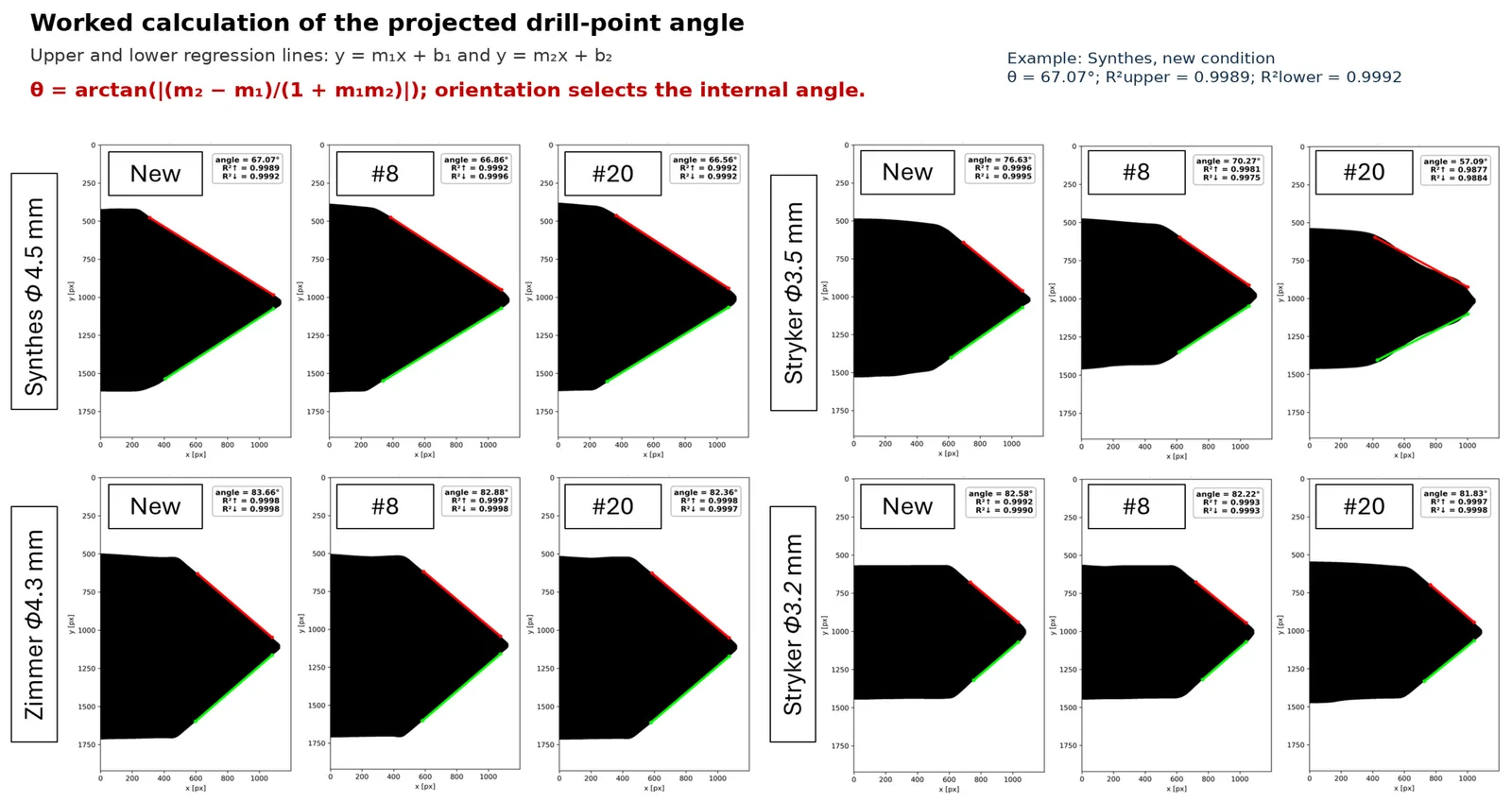
Worked example of projected point-angle calculation. The red segment is the selected linear fit to the upper silhouette flank, and the green segment is the selected linear fit to the lower silhouette flank. For slopes *m*_1_ and *m*_2_, the acute line angle is *arctan*(|(*m_2_* − *m_1_*)/(1 + *m*_1_*m*_2_)|), and line orientation selects the internal angle. For the representative new Synthes drill, the calculated angle was 67.07 degrees, with R^2^ values of 0.9989 and 0.9992 for the upper and lower fits. The remaining panels show the same procedure at the predefined stages.

**Figure 5:**
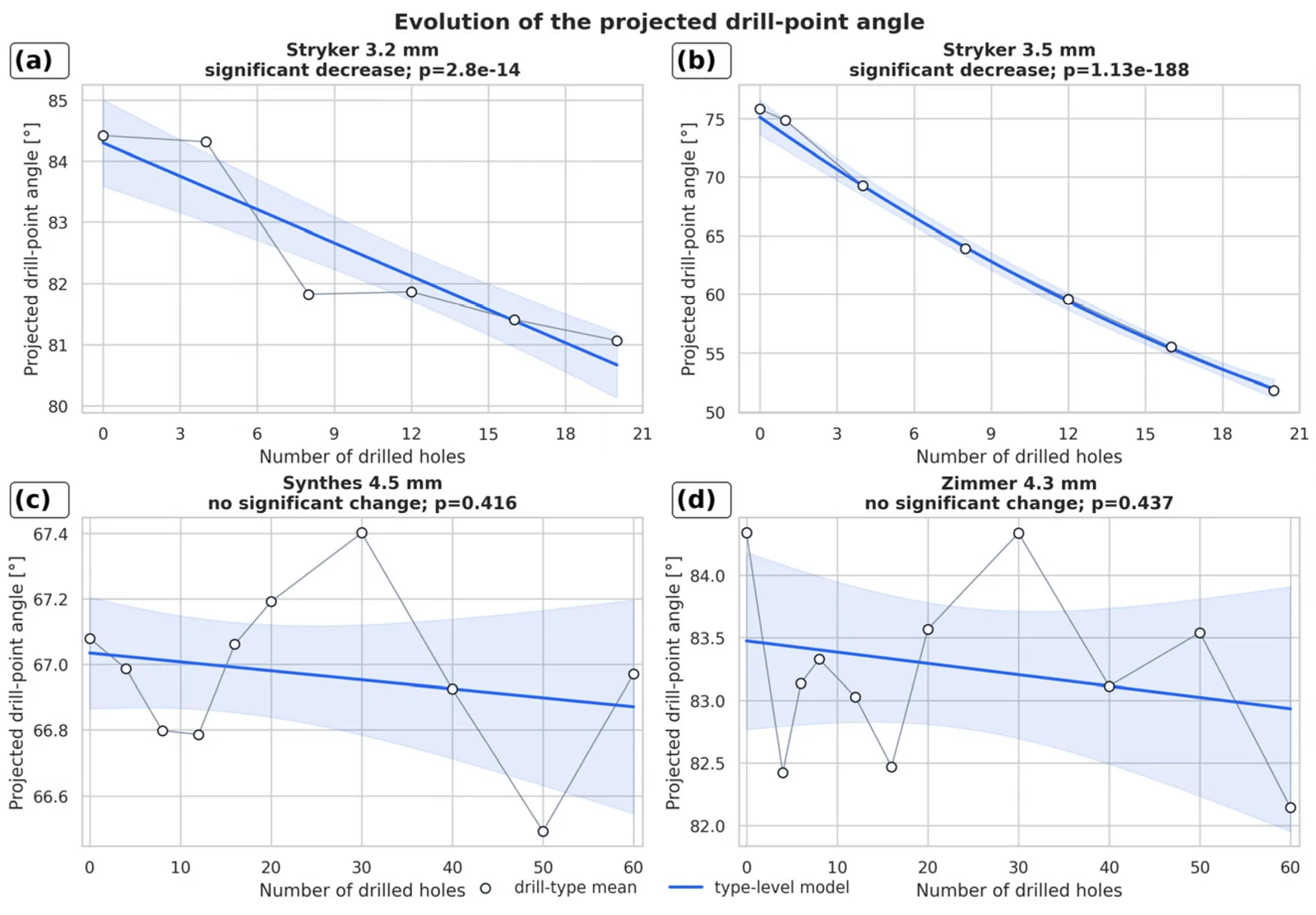
Evolution of projected drill-point angle: (**a**) Stryker 3.2 mm, (**b**) Stryker 3.5 mm, (**c**) Synthes 4.5 mm, and (**d**) Zimmer 4.3 mm. Open circles are means of four physical drills at each measured stage; blue curves are selected type-specific models; shaded regions are 95% confidence intervals for the fitted type-level mean. Point angle was measured intermittently, which explains the discrete stage trajectories in panels (**c**,**d**).

**Figure 6:**
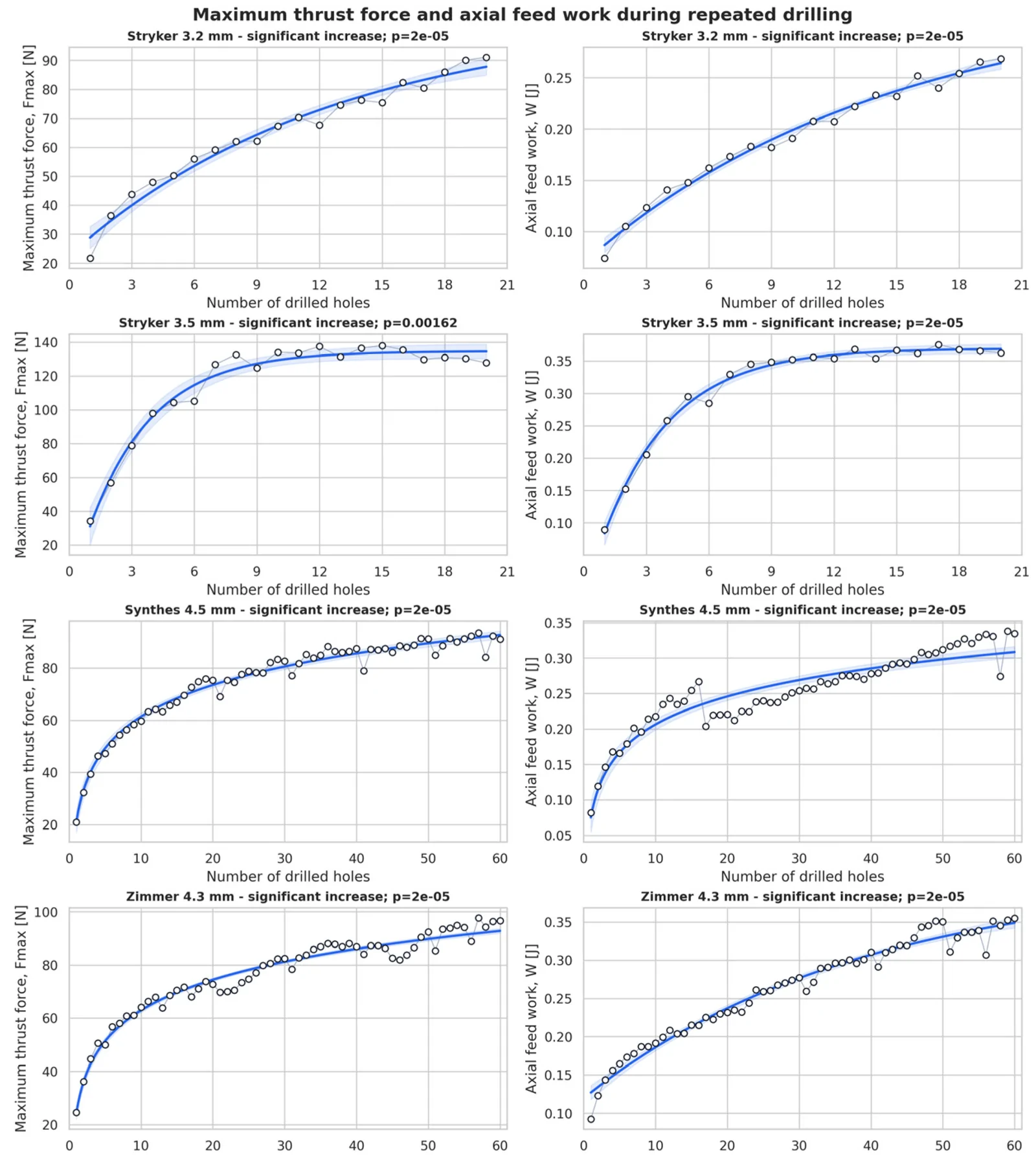
Maximum thrust force (**left column**) and axial feed work (**right column**) during repeated drilling. From top to bottom: Stryker 3.2 mm, Stryker 3.5 mm, Synthes 4.5 mm, and Zimmer 4.3 mm. The horizontal axis in every panel is the number of holes drilled by the same physical tool. Each open circle is the mean of four single-hole measurements, one per physical drill; blue curves are the selected drill-type-specific models, and shaded regions are 95% confidence intervals for the fitted type-level mean. Panels are interpreted within drill type only.

**Figure 7:**
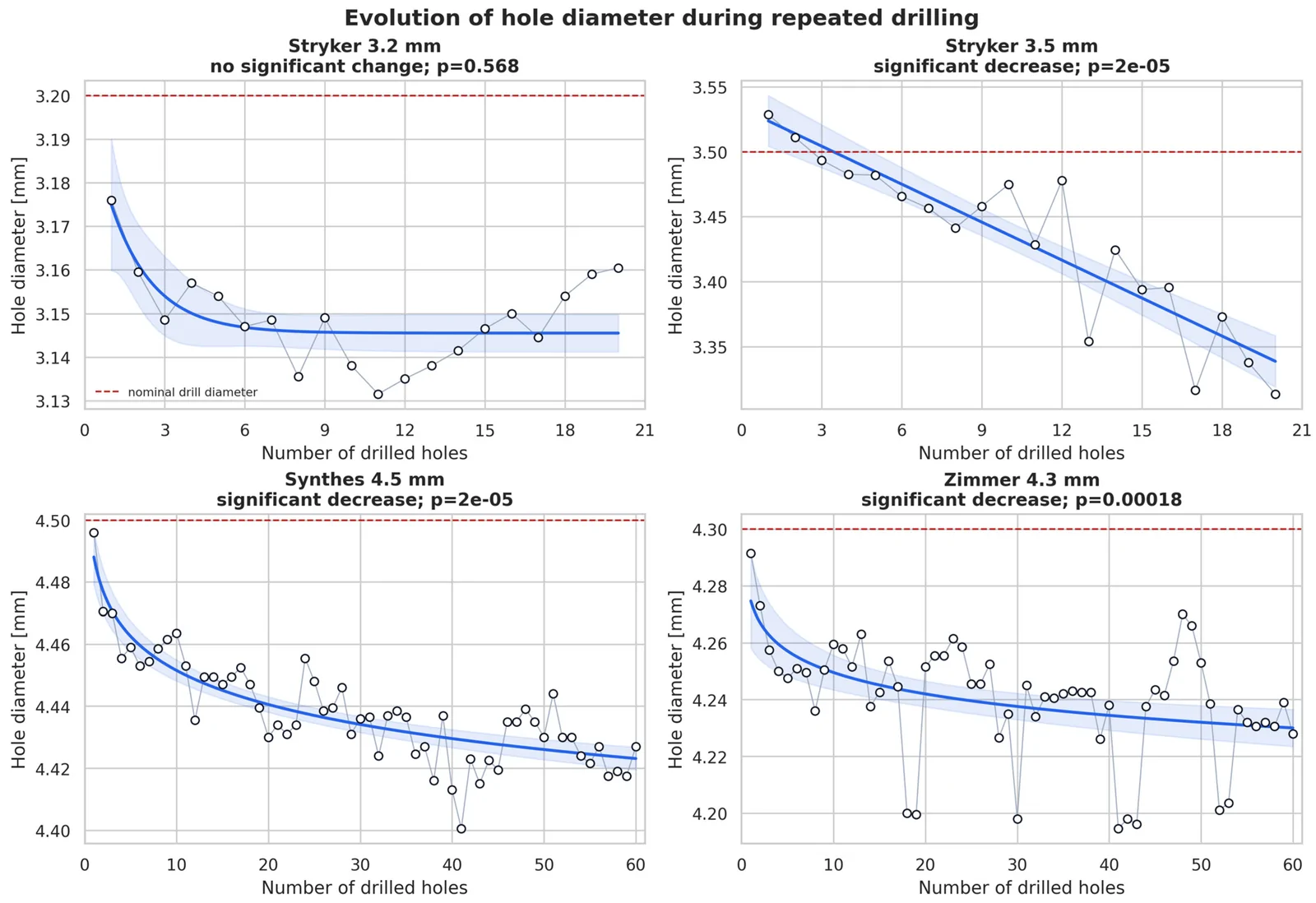
Entrance-hole diameter versus the number of holes drilled by the same physical tool. Panels show Stryker 3.2 mm, Stryker 3.5 mm, Synthes 4.5 mm, and Zimmer 4.3 mm. Each open circle is the mean of four single-hole measurements, one per physical drill; blue curves are the selected drill-type-specific models; shaded regions are 95% confidence intervals for the fitted type-level mean. The red dotted line is the nominal drill diameter, not a between-type comparator.

**Figure 8:**
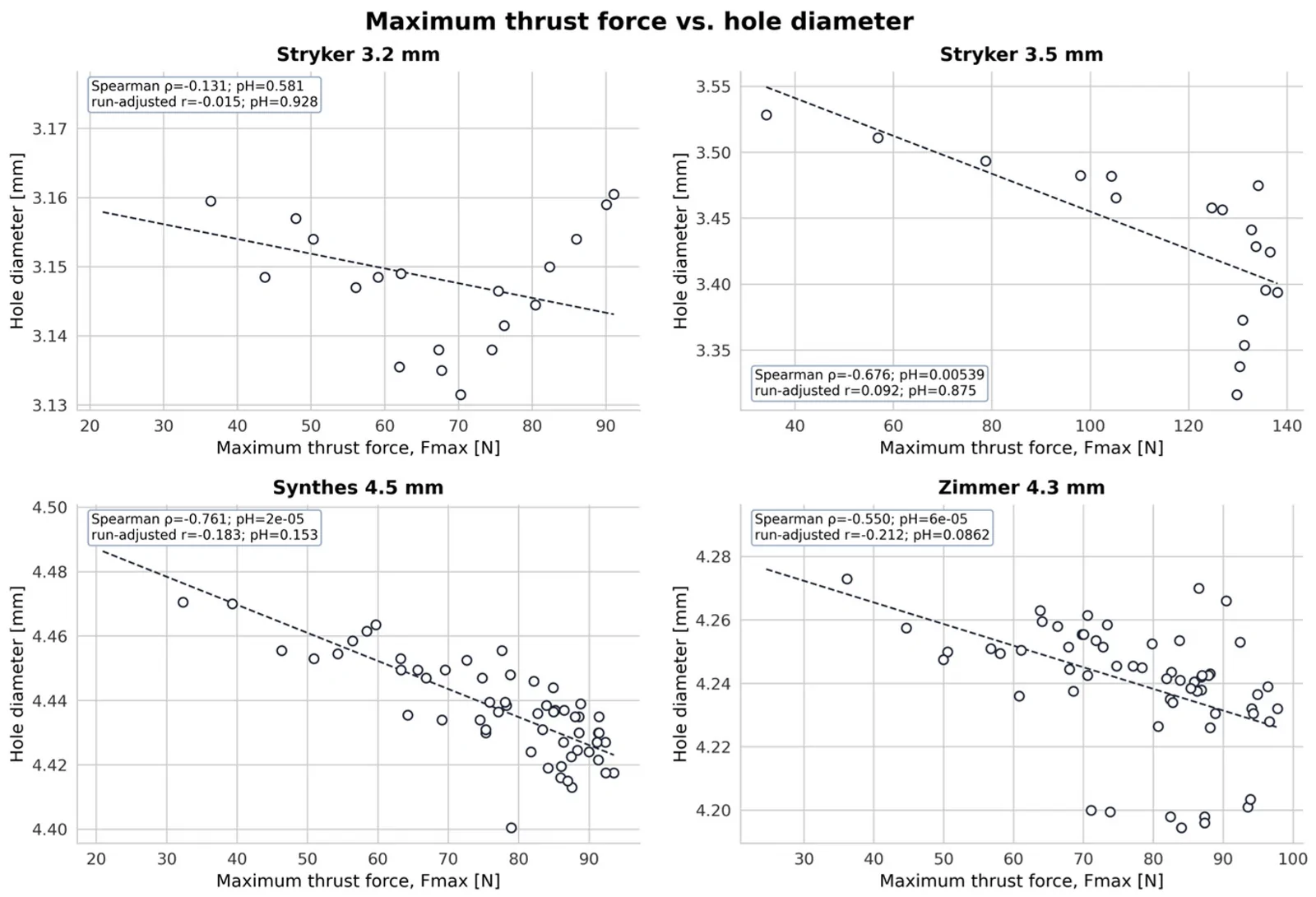
Maximum thrust force versus entrance-hole diameter at matched stages. The horizontal axis is filtered maximum thrust force, Fmax (N), and the vertical axis is entrance-hole diameter (mm). Open circles are drill-type means at common stages; dashed lines are descriptive fits.

**Figure 9:**
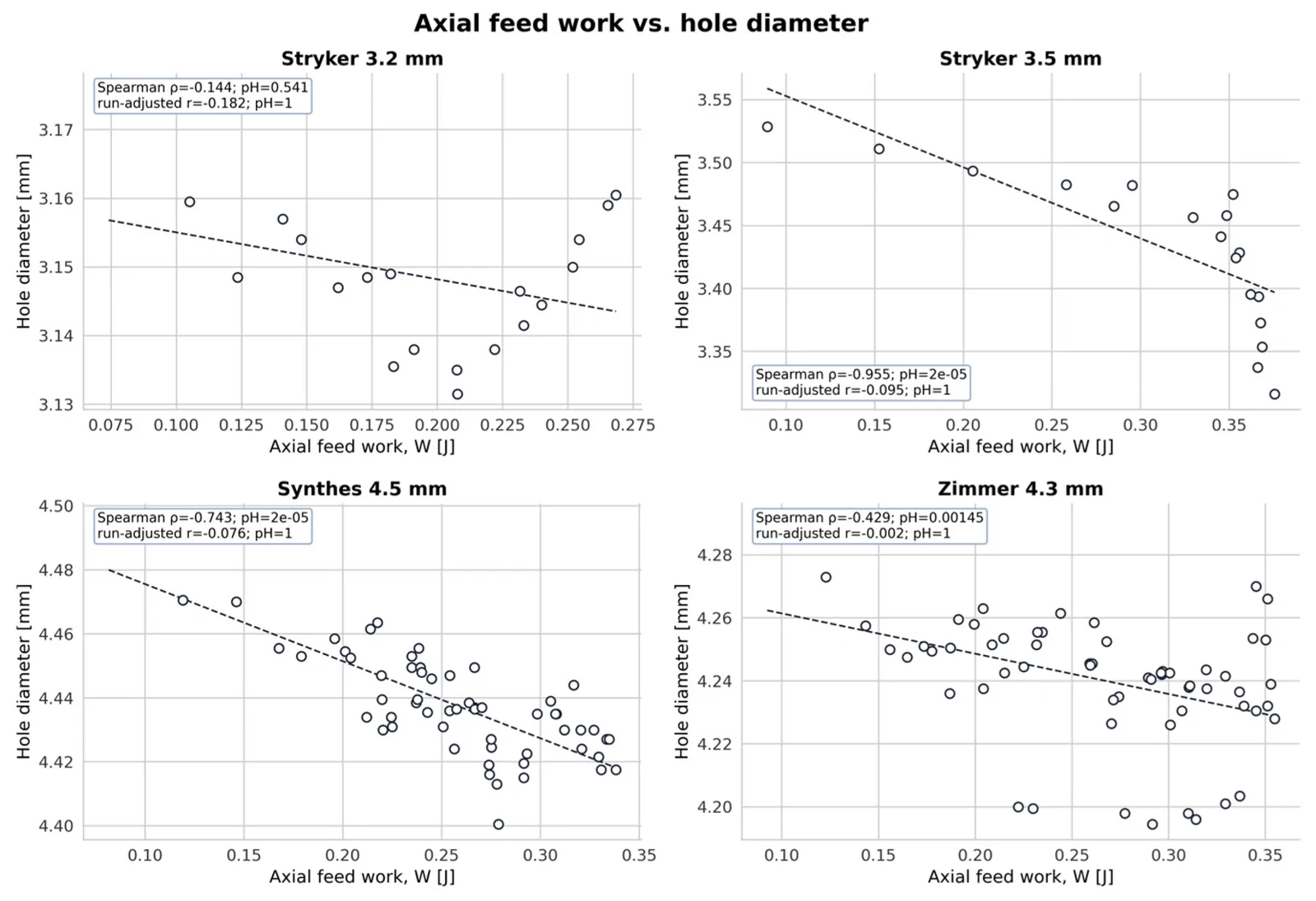
Axial feed work versus entrance-hole diameter at matched stages. The horizontal axis is axial feed work, *W* (J), and the vertical axis is entrance-hole diameter (mm). Open circles represent drill-type means at matched stages, and dashed lines represent descriptive linear fits. Unadjusted Spearman correlations are shown together with hole-number- and drill-adjusted partial associations.

**Figure 10:**
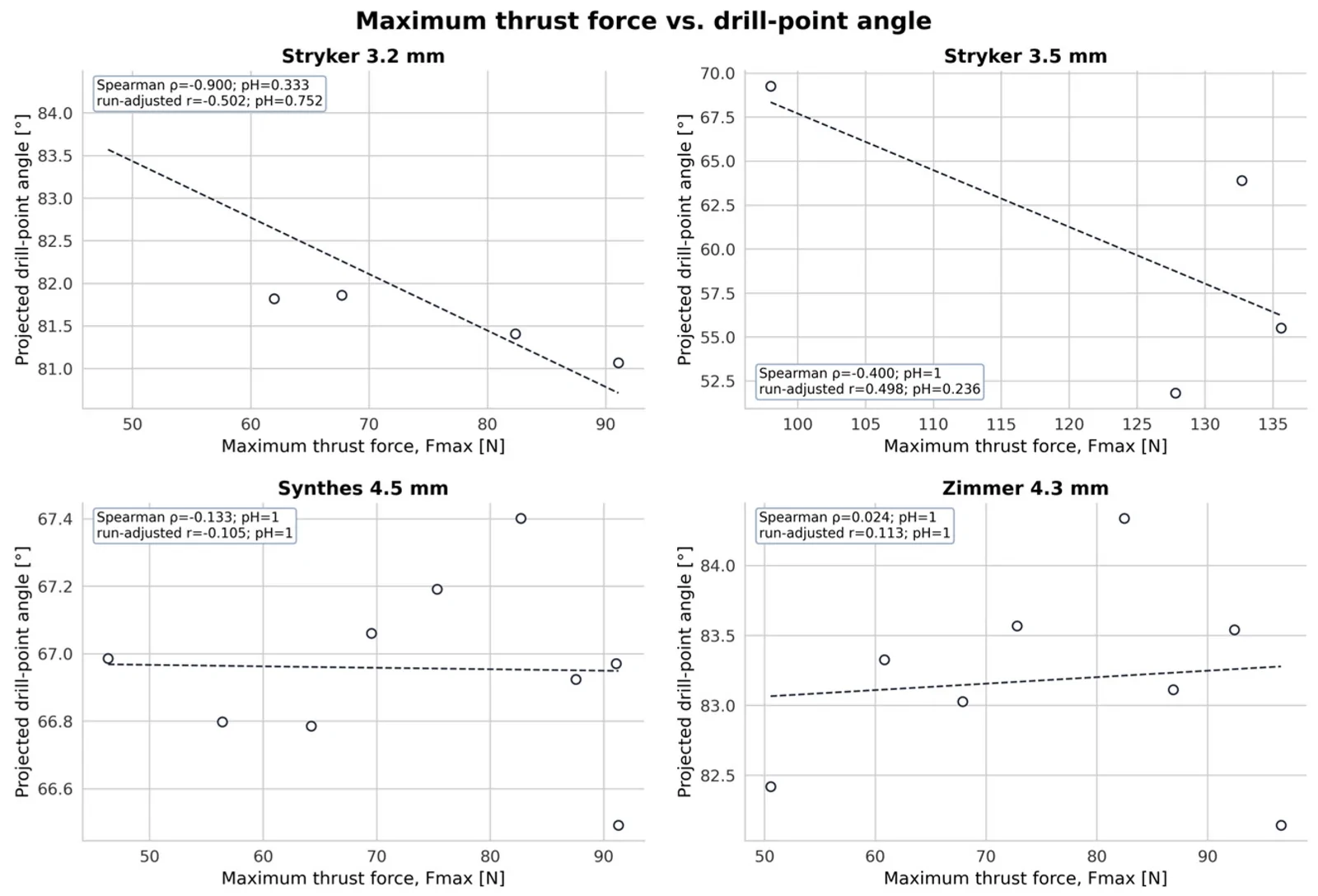
Filtered maximum thrust force versus projected drill-point angle at matched stages. The horizontal axis is F_max_ (N), and the vertical axis is projected point angle (degrees). Open circles represent drill-type means at matched stages, and dashed lines represent descriptive linear fits.

**Figure 11:**
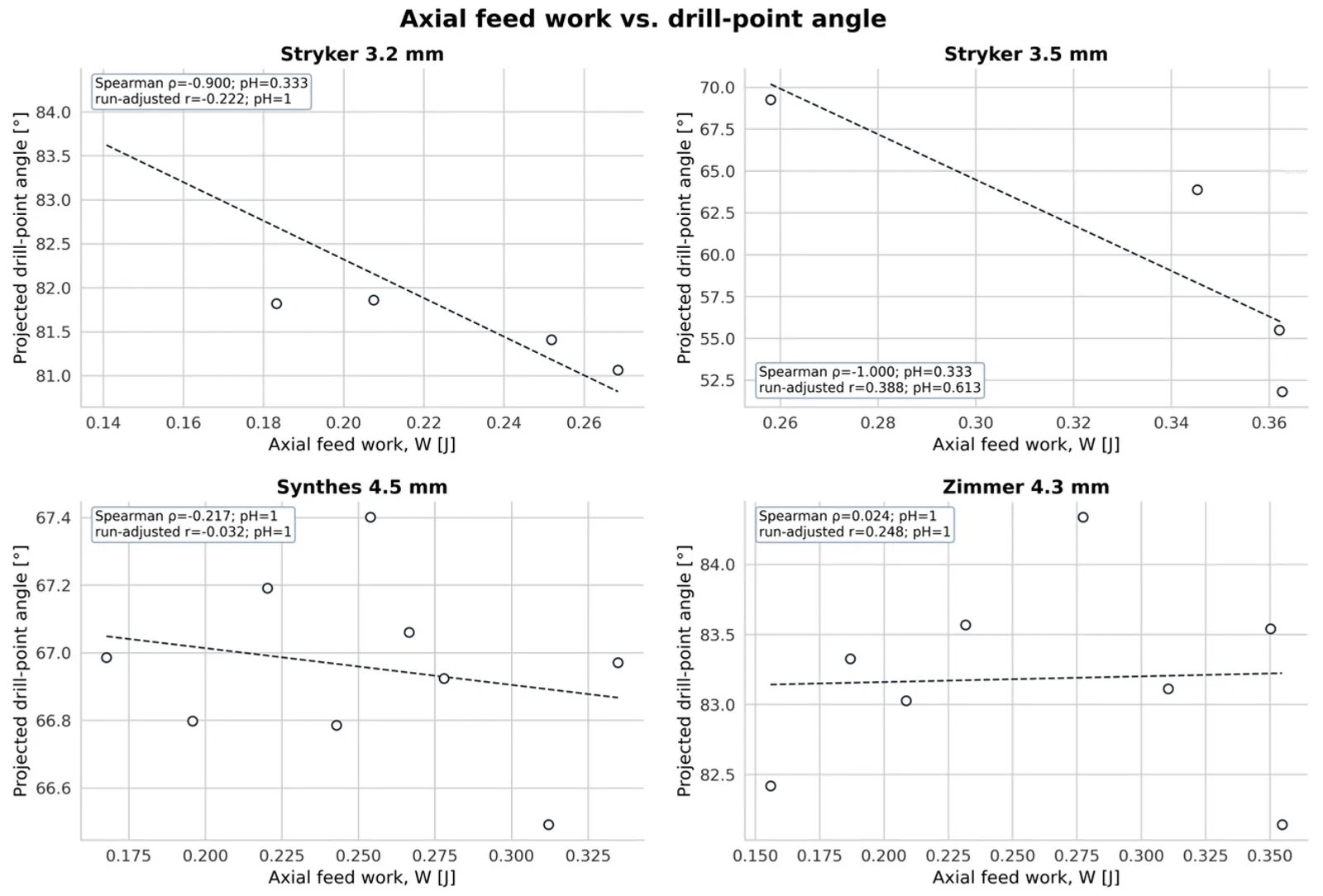
Axial feed work versus projected drill-point angle at matched stages. The horizontal axis is W (J), and the vertical axis is projected point angle (degrees). Open circles represent drill-type means at matched stages, and dashed lines represent descriptive linear fits.

**Figure 12:**
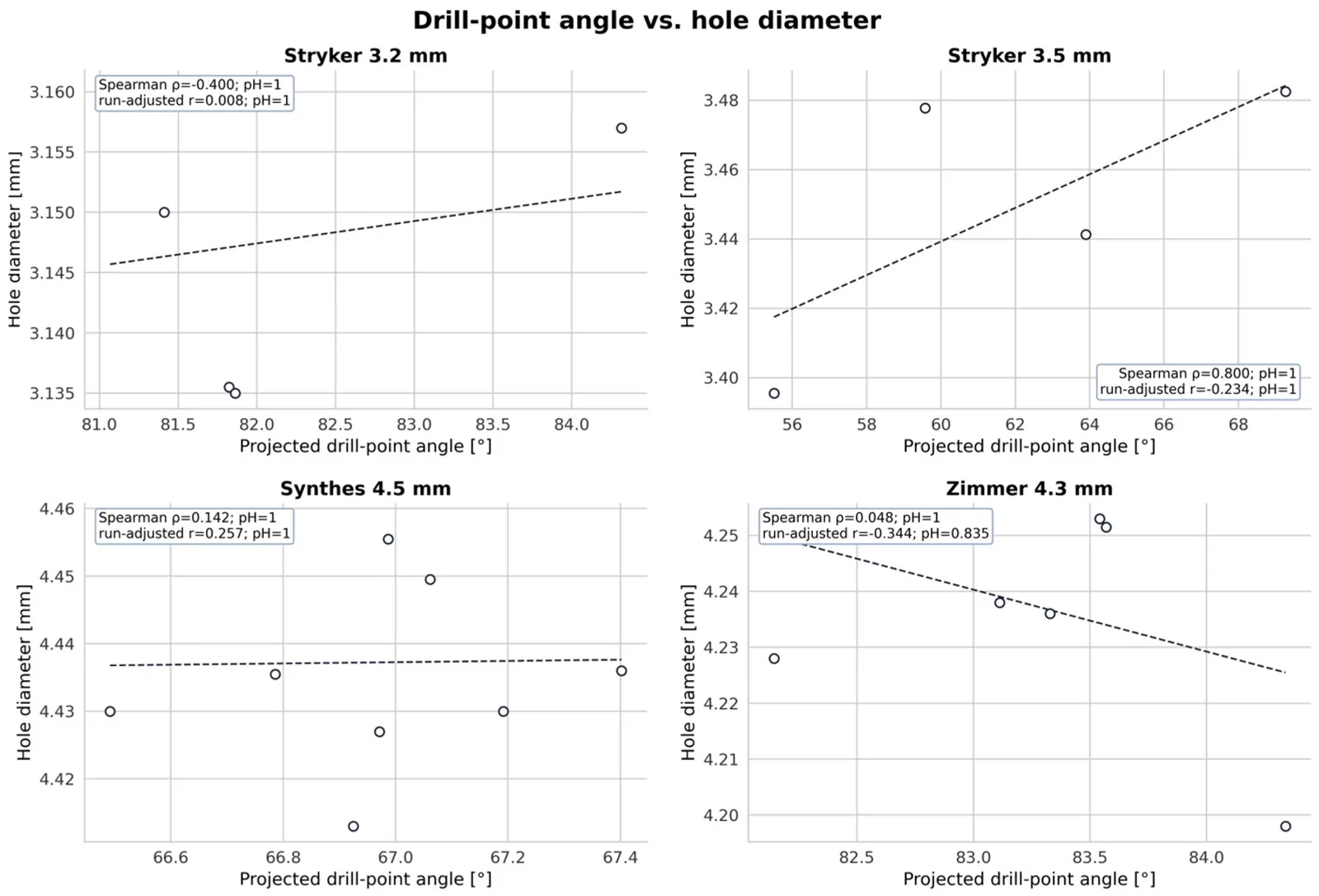
Projected drill-point angle versus entrance-hole diameter at matched stages. The horizontal axis is projected point angle (degrees), and the vertical axis is entrance-hole diameter (mm). Open circles represent drill-type means at matched stages, and dashed lines represent descriptive linear fits.

**Table 1:** Identification and nominal specifications of the investigated drill configurations.

Manufacturer	Catalog No.	Diameter mm	Overall Length mm	Product and Coupling	No. of Flutes
Stryker	700358	3.2	145	Drill bit; AO	2
Stryker	703694	3.5	122	VariAx 2 overdrill; AO	2
DePuy Synthes	310.440	4.5	145	Drill bit; quick coupling	2
Zimmer Biomet	02.00024.002	4.3	195	NCB drill bit; quick coupling	2

**Table 2:** Restricted Fe-Cr-Mo-Ni-Mn elemental profiles determined by EDXRF and exploratory compositional similarities. Values are means +/− standard deviations from five surface measurements per drill specimen; the similarities are not certified alloy identifications or measures of wear resistance.

Drill	Fe (%)	Cr (%)	Mo (%)	Ni (%)	Mn (%)	Exploratory Compositional Similarity (Non-Definitive)
Stryker 3.2 mm	81.99 ± 0.52	16.83 ± 0.52	0.493 ± 0.014	0.178 ± 0.012	0.502 ± 0.026	AISI 440-family similarity; exact subtype indeterminate
Stryker 3.5 mm	79.84 ± 0.85	11.19 ± 0.43	0.052 ± 0.002	8.832 ± 0.419	0.080 ± 0.036	Custom 455/UNS S45500-type similarity; unconfirmed
Synthes 4.5 mm	81.44 ± 0.69	17.42 ± 0.70	0.440 ± 0.011	0.332 ± 0.027	0.373 ± 0.058	AISI 440-family similarity; exact subtype indeterminate
Zimmer 4.3 mm	80.39 ± 0.94	17.65 ± 0.80	1.002 ± 0.050	0.319 ± 0.035	0.645 ± 0.086	X90CrMoV18/1.4112-type similarity; unconfirmed

Note: The EDXRF configuration quantified only Fe, Cr, Mo, Ni, and Mn, normalized within this restricted analytical system. Carbon, vanadium, copper, titanium, and niobium were not quantified. The suggested similarities are therefore exploratory and cannot establish alloy grade, heat treatment, hardness, microstructure, or wear resistance.

## Data Availability

The original contributions presented in this study are included in the article/Supplementary Material. Further inquiries can be directed to the corresponding author.

## Supplementary Materials

The following supporting information can be downloaded at: https://www.mdpi.com/article/10.3390/ma19194042/s1, Supplementary Materials contain projected drill-point angle, maximum thrust force, axial feed work, and entrance-hole diameter for the investigated drill specimens and drilling stages.
